# Asthma in Canada: perceptions and behaviours

**DOI:** 10.1186/1710-1492-7-S2-A21

**Published:** 2011-11-14

**Authors:** Harold Kim, Paolo Renzi, Malcolm Sears

**Affiliations:** 1 Department of Medicine, University of Western Ontario, London, ON, Canada and Department of Medicine, McMaster University, Hamilton, ON, Canada; 2 Department of Medicine, University of Montreal, Montreal, QC, Canada; 3 Department of Medicine, McMaster University, Hamilton, ON, Canada

## Background

The EUCAN AIM study was conducted to explore perceptions, behaviors and recent trends in asthma in 6 countries including Canada.

## Methods

Households were sampled by random dialing to identify adults and adolescents who had been diagnosed with asthma and had an asthma attack or symptoms in the past year or currently used asthma medications.

## Results

In Canada, 7,405 households were screened and 401 (363 adults and 38 adolescents) asthmatics identified. Daytime, night-time and exercise symptoms were reported every day or most days in the past 4 weeks by 29%, 9% and 16% respectively. Over the past year, 49% had shortness of breath while sitting, 30% had episodes limiting their speech and 31% woke frequently. With flares, 45% saw a physician, 28% had an ER or unscheduled visit, while 30% took oral corticosteroids in the past year. One in ten felt their life was in danger from asthma. Despite this, 77% felt asthma was completely or well controlled. Controller medications were taken as needed by 18% and not used at all by 19%. Expectations were low as asthma was considered well controlled by 60% if they had only one ER visit per year, by 62% if they had 3-4 exacerbations per year and by 44% if relievers were needed 3 times per week.

## Conclusions

This survey suggests that inadequate control of asthma persists in Canada. Perceptions and behaviors of patients are not in line with current recommendations on asthma control and treatment.

